# Estimating the Distribution of True Rates of Visual Field Progression in Glaucoma

**DOI:** 10.1167/tvst.13.4.15

**Published:** 2024-04-09

**Authors:** Giovanni Montesano, David P. Crabb, David M. Wright, Alessandro Rabiolo, Giovanni Ometto, David F. Garway-Heath

**Affiliations:** 1 City, University of London, Optometry and Visual Sciences, London, UK; 2 NIHR Biomedical Research Centre, Moorfields Eye Hospital NHS Foundation Trust and UCL Institute of Ophthalmology, London, UK; 3 Centre for Public Health, Queen's University Belfast, ICSA, Royal Victoria Hospital, Belfast, Northern Ireland, UK; 4 Department of Health Sciences, University of Eastern Piedmont “A. Avogadro,” Novara, Italy; 5 Ophthalmology Unit, University Hospital Maggiore della Carità, Novara, Italy

**Keywords:** visual field (VF), perimetry, glaucoma, progression, hierarchical modeling

## Abstract

**Purpose:**

The purpose of this study was to estimate the distribution of the true rates of progression (RoP) of visual field (VF) loss.

**Methods:**

We analyzed the progression of mean deviation over time in series of ≥ 10 tests from 3352 eyes (one per patient) from 5 glaucoma clinics, using a novel Bayesian hierarchical Linear Mixed Model (LMM); this modeled the random-effect distribution of RoPs as the sum of 2 independent processes following, respectively, a negative exponential distribution (the “true” distribution of RoPs) and a Gaussian distribution (the “noise”), resulting in a skewed *exGaussian* distribution. The *exGaussian*-LMM was compared to a standard Gaussian-LMM using the Watanabe-Akaike Information Criterion (WAIC). The random-effect distributions were compared to the empirical cumulative distribution function (eCDF) of linear regression RoPs using a Kolmogorov-Smirnov test.

**Results:**

The WAIC indicated a better fit with the *exGaussian*-LMM (estimate [standard error]: 192174.4 [721.2]) than with the Gaussian-LMM (192595 [697.4], with a difference of 157.2 [22.6]). There was a significant difference between the eCDF and the Gaussian-LMM distribution (*P* < 0.0001), but not with the *exGaussian*-LMM distribution (*P* = 0.108). The estimated mean (95% credible intervals, CIs) “true” RoP (−0.377, 95% CI = −0.396 to −0.359 dB/year) was more negative than the observed mean RoP (−0.283, 95% CI = −0.299 to −0.268 dB/year), indicating a bias likely due to learning in standard LMMs.

**Conclusions:**

The distribution of “true” RoPs can be estimated with an *exGaussian*-LMM, improving model accuracy.

**Translational Relevance:**

We used these results to develop a fast and accurate analytical approximation for sample-size calculations in clinical trials using standard LMMs, which was integrated in a freely available web application.

## Introduction

Glaucoma is an optic neuropathy characterized by progressive damage to the optic nerve head and damage to the visual field (VF). Loss of VF results from damage and death of the retinal ganglion cells, the axons of which converge on the optic nerve head (ONH) and are damaged as the disease progresses. VF progression is monitored clinically through standard automated perimetry (SAP), a test during which patients are asked to fixate on a central target while light stimuli of varying intensity are presented at different locations in their VF. Patients are asked to respond by pressing a button every time a stimulus is seen to map their sensitivity across the VF.

Changes in the VF are quantified in a variety of ways. One common method is to calculate the rate of progression (RoP) of a global index of VF sensitivity, such as the mean deviation (MD), over time through linear regression. This method is commonly used in clinical care and has been proposed for randomized clinical trials (RCTs).^1^^–^^4^ Because of the progressive and irreversible nature of the disease, VF sensitivity is only expected to worsen, resulting in a negative RoP, or remain unchanged over time. However, when the RoP is calculated through linear regression, the distribution of RoPs in clinical populations^5^^–^^11^ is negatively skewed, but many patients show a positive RoP. Finding methods to estimate the underlying distribution of “true” RoPs would help the interpretation of the results of population-based studies and, importantly, of RCTs quantifying the effect of different treatment strategies.

**Figure 1.**
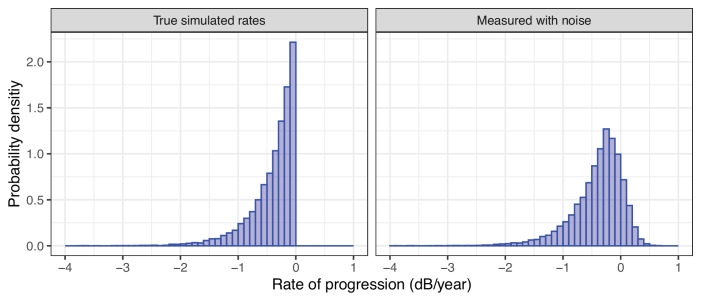
Simulated examples, 20,000 eyes, and 11 tests over 10 years. The histogram of the true rates of progression (RoPs) of the mean deviation (MD) in the simulation is reported on the left. On the *right*, the distribution of the RoPs estimated with simple linear regression in the presence of Gaussian noise on the MD (standard deviation = 2 dB). The *bars* represent the estimated probability density, that is, the total area under the histogram is normalized to 1.

A few attempts have been made to describe this distribution analytically. Andrew Anderson^9^^,^^10^ proposed a hypergeometric-secant distribution for the RoPs in a clinical glaucoma cohort. Zhang et al.^11^ and Swaminathan et al.^12^ used a log-Gamma distribution for a similar purpose in their modeling of VF progression, where the log-Gamma distribution was used to model the distribution of the random effects in a hierarchical linear mixed effect model (LMM). Although these distributions fit the data by capturing some essential features (mainly, the skewness), they fail to give a description for a plausible underlying process of VF progression. In fact, a purely negative distribution of true RoPs is expected to arise to a negatively skewed distribution with positive values when measured in the presence of noise (see an example in Fig. 1). Additional factors, such as patients’ learning,^13^ can contribute to the presence of observed positive RoP slopes and contaminate the true distribution. This relationship between true distribution and its noisy estimate from data has been already highlighted with simulations by Andrew Anderson.^9^

In this work, we model, with few assumptions, the underlying distribution of true RoP slopes, isolating the effect of measurement noise and learning. This method is based on hierarchical modelling of the RoP of MD in a clinical population. We validate our method in a large cohort of eyes with long test series. We then provide a simple analytical description of the resulting distribution and show how this can be used to model a treatment effect in simulations. We finally show how this description can be used to calculate analytical power curves for LMMs for a variety of clinical trial designs, without making use of time-consuming simulations.^2^^,^^4^

## Methods

### Clinical Database

This database has been previously described elsewhere. VF data were extracted from an electronic medical record (Medisoft; Medisoft Ltd., Leeds, UK) from five National Health Service Hospital Trust glaucoma clinics in England in November 2015.^14^^–^^16^ All patient data were anonymized upon data extraction and transferred to a single secure database at City, University of London. Subsequent analyses of the data were approved by a research ethics committee of City, University of London. The study adhered to the Declaration of Helsinki and the General Data Protection Regulation of the European Union. The VFs included in this analysis were 24-2 tests performed with an Humphrey Field Analyzer (HFA), Goldmann III stimulus size and the Swedish Interactive Testing Algorithm (SITA Standard or SITA Fast). The whole database was composed of 576,615 VFs from 71,361 people, obtained between April 2000 and March 2015. VFs with a percentage of false positive errors ≥ 15% were excluded. No exclusion criteria were applied on fixation losses or false negative errors, following evidence from the literature.^17^^,^^18^ Additional metrics, such as gaze tracking data, were not available in this dataset and could not be used to assess reliability. We included all patients with at least 10 VF tests performed over at least 4 years in one or both eyes and an MD worse than −2 decibels (dB) in at least 2 (not necessarily consecutive) VFs^19^^–^^21^ in the same eye. This was used as a surrogate for the diagnosis of glaucoma, in the absence of a definitive label. Subjects with this level of damage and length of follow-up were likely to be either strong glaucoma suspects or persons with glaucomatous optic neuropathy. However, VF loss from other causes, such as vascular or neurological issues, could not be definitively excluded. VFs performed after any ocular surgery other than cataract were also excluded. Finally, only one eye from each patient was selected, at random if both were eligible, leaving 44,371 VFs from 3352 eyes. Because only one eye was included, the term eye and subject will be used interchangeably. This set of eyes has been used previously for other analyses.^4^^,^^22^

**Figure 2.**
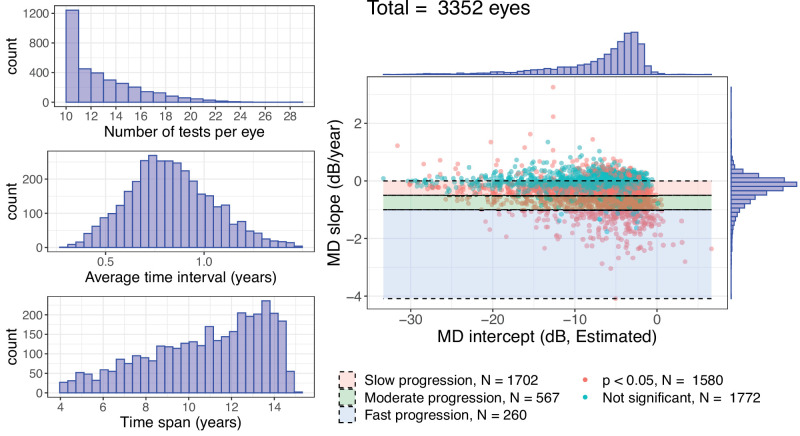
Descriptive plots of the dataset used in this study. The intercepts and rates of progression are estimated through linear regression. The average time interval is the average time difference between pairs of consecutive tests in each series. The time span is the overall time length of each series. Mean deviation (MD) intercepts, slopes, and *P* values were calculated through ordinary least square regression. Cutoff values for moderate and fast progression were −0.5 dB/year and −1 dB/year, respectively. Significant progression was defined as a negative MD slope with a *P* value < 0.05.

Patients’ demographics were (median and interquartile range [IQR]): baseline age 68 years (IQR = 60 to 75 years); average best corrected visual acuity (BCVA) 0 (IQR = −0.1 to 0.2) logMAR; average intraocular pressure (IOP) 16 (IQR = 14 to 18) mm Hg; average MD −6.44 (IQR = −11.06 to −4.07) dB; average pattern standard deviation 5.68 (IQR = 3.27 to 9.06) dB. Average values were calculated from all the available data recorded during the time frame of the tests. The median length of follow-up was 11 years (IQR = 8 to 13 years) and the number of VFs per series was 12 (IQR = 11 to 15). Descriptive plots are reported in Figure 2.

### Hierarchical Modeling of Progression

#### Model for the Slope

For our modeling, we assumed that the “true” underlying linear RoP for the MD could take values that were either zero or negative. Most distributions with a positive support for their probability distribution function (PDF) can describe such a phenomenon with a simple sign reversal. We chose the exponential distribution because of its plausibility and simplicity (it is described by a single parameter, λ, see Appendix). As shown in Figure 1, a set of “true” exponentially distributed RoPs will exhibit the typical two-tailed skewed distribution seen in clinical datasets when estimated with linear regression in the presence of noise. When the noise is normally distributed, the resulting distribution is an exponentially modified Gaussian distribution (*exGaussian*). The formula for the PDF and the cumulative density function (CDF) of this distribution are reported in the Appendix. In simpler terms, each observed RoP can be thought of as the sum of a random draw from two independent distributions, an exponential (the “true” rate) and a Gaussian (the noise). Therefore, the mean of the resulting *exGaussian* distribution is equal to the sum of the means of the two underlying exponential and Gaussian distributions. Similarly, the *exGaussian* variance is equal to the sum of the two variances. Note that the PDF of the distribution of the sum of two random variables is obtained as the convolution of their PDFs. The *exGaussian* PDF is the convolution of a Gaussian and exponential PDFs.

In this application, the standard deviation (SD) of the Gaussian noise is the standard error (SE) of the linear regression slope, which is determined by the number of observations (i.e. tests), the variance of the independent variable (i.e. time), and the variance of the dependent variable around the prediction, that is, the noise of the MD, assumed Gaussian. This latter assumption ensures that the noise distribution of the slope is also Gaussian, even for small sample sizes, that is, a small number of tests in the series.

#### Model for the Intercept

A model for the intercept for linear MD progression cannot be as easily constrained as the model for the slope. Visual inspection of the distribution of the empirical intercepts (see Fig. 2) would suggest a distribution similar to that of the slopes. Although the exact distribution cannot be an *exGaussian* (for reasons that will be explained), it is useful to understand whether it can be a practical approximation.

In an ideal and simplified scenario, all patients start with a “healthy” MD of exactly 0 dB (i.e. at the exact expected sensitivity values for age-matched healthy subjects), progress with exponentially distributed RoPs and all have their first VF test at the same time from the development of glaucoma (for example, 5 years). In this case, the “true” distribution of baseline MDs will also be exponential, because it would result from the product of the RoPs and the interval to first detection (i.e. RoP dB/year × 5 years in this example). However, there are at least three components of noise that can contaminate this ideal scenario. One is the measurement noise, reflected in the SE of the intercept, similarly to the slope. This would produce an *exGaussian* distribution if the error is assumed Gaussian. The second component is intersubject variability, that is, not all patients have a “true” MD of 0 dB before developing glaucoma. If the distribution of “true” healthy MD values is also Gaussian, adding this element of noise would also result in an *exGaussian* distribution. Finally, a third component is the actual time interval between the development of glaucoma and the first VF test. This is obviously unlikely to be the same for all subjects, as assumed in the ideal example. This interval can be described by a strictly positive random variable, such as one following a *Gamma* distribution. The product of a Gamma and an exponential distribution is not an exponential distribution,^23^ but would still result in a single-tailed negative distribution which can be approximated with an exponential distribution with mean equal to the product of the means of the original exponential and Gamma distributions. The exact PDF for the product distribution can be expressed analytically, but its parameters are difficult to reliably estimate, especially in the presence of additional Gaussian noise. A comparison between the exponential approximation and the exact PDF in a simulated example is provided in the Appendix.

From the above, although certainly not a “correct” description, an *exGaussian* distribution for the intercept appears to be a reasonable approximation. For our main analyses, we chose a simplified model that estimated the Gaussian noise only from the SE of the intercept (see the next paragraph). An alternative model, that included the effect of intersubject variability, was also tested, but did not provide any improvement, because the estimated intersubject variability converged toward zero during fitting.

#### Bayesian Hierarchical Modeling

Bayesian computation was used to estimate the parameters of a hierarchical LMM using Just Another Gibbs Sampler (JAGS^24^) within the R environment (R Foundation for Statistical Computing, Vienna, Austria). The LMM modeled the trend of MD over time, with two levels in the hierarchy, the population level and the subject level, because only one eye per subject was included. At the subject level, the model estimated an intercept and slope parameter for each subject. Like in standard Gaussian-LMMs, the response variable was assumed to be normally distributed and homoscedastic around the predicted value. This is a common assumption, although not necessarily accurately reflective of the data.^25^ The choice of a Gaussian random effect distribution for the standard LMM does not have a strong theoretical justification, other than its relative simplicity, generalizability, and widespread use in the literature.^1^^,^^2^^,^^4^^,^^12^^,^^22^^,^^26^^–^^29^

The intercept and slope for each subject were modeled as random effects and sampled from a population level distribution (higher level of the hierarchy). The random intercepts and slopes followed an *exGaussian* distribution. Instead of defining the *exGaussian* PDF in the model, Bayesian computation allowed us to replicate the process underlying the generation of the observed data. This was achieved by sampling values from the exponential and Gaussian distribution separately and modeling the observed slope as the sum of the two values.

The Bayesian procedure estimated, among other population parameters, the parameter λ of the exponential distributions and the mean of the Gaussian noise for the slopes and intercepts. The SD of the Gaussian noise was not estimated, but rather calculated from the estimated residual SE for the MD (σ_e_, i.e. the MD noise at the subject level). More details on the implementation of the Bayesian model are reported in the Appendix. One important aspect to note is that each subject in the dataset had a variable duration of follow-up time and number of tests. This introduced variation in the SE of the slope across subjects. Deriving the parameter sigma from the SE of the residuals allowed us to account for this, by calculating the expected SE of the intercept and slope for each subject (see Appendix). The data were also modeled with a standard Bayesian hierarchical LMM, using a Gaussian distribution for both intercepts and slopes. The two models were compared using the Wantanabe Akaike Information Criterion (WAIC) as implemented in the *loo* package for R.^30^ Note that, because of our specification of the *exGaussian*-LMM, the number of estimated parameters is the same for both models, because the *exGaussian*-LMM does not require an estimation of the between-subject level variance (see Appendix). This is, in fact, given by the sum of the variance of the exponential distribution (which is simply 1/λ^2^) and the variance of the Gaussian noise distribution (derived from the residual SE). However, the WAIC is influenced by the choice of prior distributions, which were partially constrained in our *exGaussian*-LMM to improve stability with smaller datasets and shorter VF series (see Appendix). The model comparison was therefore based on a version of the *exGaussian*-LMM for which the prior distributions were practically uninformative, so that the prior information was similar to that provided for the standard Gaussian-LMM (see Appendix). An additional comparison was performed by evaluating the empirical CDF of the RoPs and intercepts against the CDF of the estimated Gaussian and *exGaussian* distributions with a Kolmogorov-Smirnov test.^31^

We hypothesized that the mean of the Gaussian noise for the RoP would capture the effect of learning, which would effectively introduce a positive offset on the estimates of the “true” RoPs. We tested this by fitting the *exGaussian*-LMM on VF series that were progressively trimmed at the beginning, to observe whether a positive offset in the mean would reduce by eliminating earlier tests in the series, where the effect of learning would be the greatest. We kept the minimum series length at 10 VFs, as in the original analysis. The sample size was therefore reduced at each trimming step.

#### Rate of Progression According to Baseline Visual Field Damage

We further estimated the exGaussian-LMM parameters for different levels of baseline VF loss. The cohort of patients was divided into three groups based on their estimated baseline MD (average of the first 2 tests): early (baseline MD ≥ −6 dB); moderate (−6 dB > baseline MD ≥ −12 dB); and advanced (baseline MD < −12 dB). Of note, the classification was applied to a cohort already selected to have at least 2 tests with an MD < −2 dB. For this analysis, the LMM was modified so that three different values of the population parameters (i.e. the exponential rate, the Gaussian mean, and the residual SE) were estimated, one for each severity group. This is equivalent to fitting the LMMs on each group separately, but obtains the parameter estimates from the same Markov Chain Monte Carlo draws (see Appendix). This allows statistical comparisons between parameters estimated from the different groups. For this comparison, we used a Bayesian equivalent of the frequentist *P* value, derived from the *P* direction.^27^^,^^28^^,^^32^ Details are reported in the Appendix.

### Simulations and Power Calculations

The population parameters estimated with the *exGaussian*-LMM were used to perform computer simulations of hypothetical treatment effects in RCTs. One advantage of our modeling is that it allows for a clear definition of a proportional treatment effect by scaling the parameter λ of the exponential distribution. This is different from previous attempts, where a change in slopes was somewhat poorly defined and achieved either by completely halting the progression of a proportion of patients^2^ or by modifying observed RoP slopes by an additive factor to achieve the desired proportional change in the average RoP.^4^ Another important difference is that previous attempts used observed regression slopes to model the “true” RoP, with simulated noise added to this “true” trend.^2^^,^^4^ However, empirically calculated slopes will also be affected by noise, as explained previously. Instead, with our method, we simulated the “true” RoP slope for each eye as a draw from the “denoised” exponential distribution of slopes.

The simulation procedure was as follows:

1. Perform a simple linear regression of MD over time for each eye on the real VF series.
2. From the linear regressions, calculate the SE of the residuals (σ_e_), an unbiased estimate of the individual variability.
3. Select a sample size (*N*) and a treatment effect (E).
4. Randomly sample, without replacement, *N* subjects for the placebo arm and *N* for the treatment arm.
5. Generate “true” RoP values from an exponential distribution with rate λ for the placebo arm and λ/(1-E) for the treatment arm.
6. For each eye, generate a synthetic linear MD series for a time vector *t* using the simulated RoP for the slope and the real baseline MD as the intercept.
7. For each eye, add Gaussian noise using the subject specific variability calculated at point (2).
8. Fit an LMM and calculate the *P* value of the interaction term between treatment and time (treatment effect) – Method 1 (see later).
9. Fit simple linear regressions to the simulated data and perform an independent sample *t*-test on the “observed” slopes comparing the two arms – Method 2 (see later).
10. Repeat 1000 times from (4) to (9) for increasing *N* and E.

A larger value for the rate λ means a faster decay of the exponential tail, that is, slower average progression in the sample (closer to 0 dB/year). The treatment effect E indicated the proportional reduction in the true RoP (e.g. a 30% neuroprotective effect would mean dividing the rate λ by 0.7). The time vector *t* indicated the time of 16 tests over 2 years (with retest sessions), replicating the testing scheme of the United Kingdom Glaucoma Treatment Study (UKGTS) trial.^33^ In reality, the variability of the MD varies with the level of damage.^25^ However, global metrics, such as the MD, are predominantly influenced by individual performance.^4^^,^^34^^,^^35^ Moreover, considering that we used the observed baseline MD as the intercept for our simulated series and that the simulated trial extended for only 2 years, it is reasonable to assume that the variability measured from each subject over at least 10 VFs (median of 11 years of follow-up) would be a realistic representation of the variability exhibited by that subject over such a hypothetical trial. Note that empirical approaches based on standardizing and permuting the observed residuals have the disadvantage of providing a biased estimate of variability (the SD of the residuals is smaller than the SE of the residuals).

#### Method 1

For each simulation, we calculated a *P* value for the difference in RoP between the two arms with an LMM using the *lme4*^36^ and *lmerTest*^37^ packages for R. The LMM had the MD as the response variable, with time (continuous) and treatment arm (factor) as fixed effect predictors. An interaction term between the treatment arm and time modeled the average difference in the RoP between the two arms. Random effects for both the intercept and the slope at the subject level were modelled as a bivariate Gaussian distribution, which included the correlation between the two parameters. Residuals were assumed conditionally independent. The *lmerTest* package offers various methods to calculate *P* values for LMMs, which differ in the way they calculate the degrees of freedom. A common choice is Satterthwaite's method, which provides an approximate t-distribution for the parameters.^37^

#### Method 2

When complete data are available for all subjects (i.e. all subjects have the same number of tests), which is a common assumption in power calculations, the *P* value obtained for LMMs with Satterthwaite's method is well approximated by that calculated from a two-sample *t*-test on the empirical RoPs obtained with simple linear regression. We confirmed this by performing a two-sample *t*-test for all our simulations. We further exploited this property to obtain analytical approximations of the LMM power curves by calculating the expected variance and mean of the empirical exGaussian RoP distributions, based on the testing schedule, the average residual SE and the simulated exponential distribution for the “true” RoP. These values for the expected mean and variance were then used to analytically calculate the power of a *t*-test for two samples with unequal variances (using the *pwr* package for R).

Note that, by construction, the distribution of the empirical slopes is in violation of the distributional assumptions for the random effects made by standard LMMs (Gaussian). However, for large enough samples, the distribution of the estimates for the fixed effect parameters is expected to converge to a Gaussian. For the simulated RCTs, the power was calculated as the proportion of simulations in which the *P* value for the treatment effect with each method was < 0.05. Confidence intervals for the power was calculated as 1.96 × SE_p_, where SE_p_ is the standard error for the probability of a binomial process, calculated as $SE_{p}=\sqrt{p_{p<0.05}*\left(1-p_{p<0.05}\right)/N}$, where *p*_*p* < 0.05_ is the proportion of significant *P* values in the simulations and *N* is the total number of simulations. Naturally, the analytical approximation has the advantage of allowing quick adjustments of the parameters to accommodate for various trial designs, expected levels of variability and neuroprotective effects. We developed an interactive web application in the Shiny environment^38^ to allow users to experiment with the effect of these parameters on the estimated power curves. The app is available at https://giovannimontesano.shinyapps.io/Sample_size/.

## Results

### Estimated Distribution of the Rates of Progression

**Figure 3.**
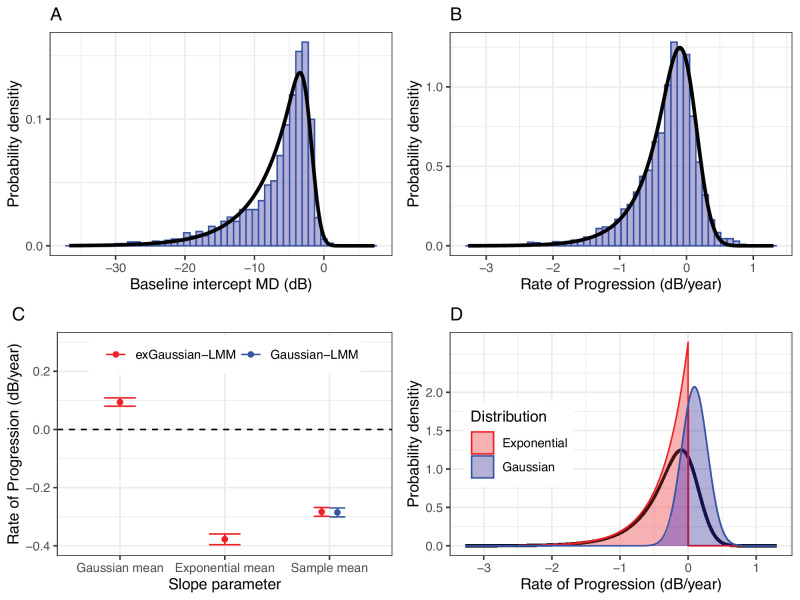
Empirical and estimated distributions for the intercepts (**A**) and slopes (**B**) in the dataset. The estimated population-level slope parameters from the exGaussian and Gaussian LMMs are shown in (**C**), with their corresponding 95% credible intervals. Note that the “sample mean” is the estimated average for the slopes. This is directly estimated by the Gaussian-LMM, but can be obtained as a sum of the exponential and Gaussian distribution means in the *exGaussian*-LMM. The exponential and Gaussian components of the *exGaussian* distribution for the slopes are reported in (**D**), together with the resulting distribution (*black curve*, obtained as the convolution of the exponential and Gaussian probability distribution functions).

The Table reports the parameters estimated by the LMMs. Figure 3 shows the estimated PDFs on top of the empirical distributions. The exponential and Gaussian components of the RoP distribution are also plotted separately for the slopes, the main parameter of interest. Note that the mean of the Gaussian noise component was close but significantly different from 0 dB/year. This would lead to biased estimates of the average RoP, resulting from the sum of the mean of the exponential distribution and the Gaussian distribution (see Fig. 3C). Importantly, this biased sum corresponds to the average RoP estimated with the Gaussian-LMM.

**Table.** Estimated Model Parameter Values for the Slope

Model	Parameter	Estimate
*ExGaussian*-LMM	Exponential mean	−0.377 [−0.396 to −0.359]
	Gaussian mean	0.094 [0.080 to 0.109]
	Gaussian variance	0.037 [0.037 to 0.038]
	Sample mean	−0.283 [−0.299 to −0.268]
Gaussian-LMM	Mean	−0.285 [−0.301 to −0.270]
	Variance	0.173 [0.163 to 0.184]

The Gaussian variance in the *exGaussian*-LMM corresponds to the average squared standard error of the slope. The exponential mean is −1/λ. The sample mean is the sum of the exponential and gaussian means. LMM, linear mixed model.

The WAIC indicated a significantly better fit with the *exGaussian*-LMM (estimate and SE 192174.4 [SE = 721.2]) than with the Gaussian-LMM (192595 [SE = 697.4327], difference 157.2 [SE = 22.6]). This was confirmed by the Kolmogorov-Smirnov (KS) test for the slopes, which showed no significant difference between the empirical CDF of the RoPs and the estimated *exGaussian* distribution (*P* = 0.108), but a highly significant difference with the estimated Gaussian distribution (*P* < 0.0001). A table comparing the WAIC for the two models for increasing series length (from 4 to 10 tests) is provided as Supplementary Material. For the distribution of the intercepts, the *exGaussian* approximation was, as expected, not as good as for the observed RoPs (KS = *P* < 0.0001). The empirical distribution showed a faster decay in the negative tail compared to the prediction. This is in agreement with theoretical expectations (see Appendix). Nevertheless, a model with a Gaussian distribution for the random effects on the intercepts provided a significantly worse fit (WAIC: 192473.3 [SE = 710.6]) compared to the full *exGaussian*-LMM (difference: 149.4 [SE = 22.4]). The random effect estimates of intercepts and slopes obtained with the two LMMs are reported as Supplementary Material.

**Figure 4.**
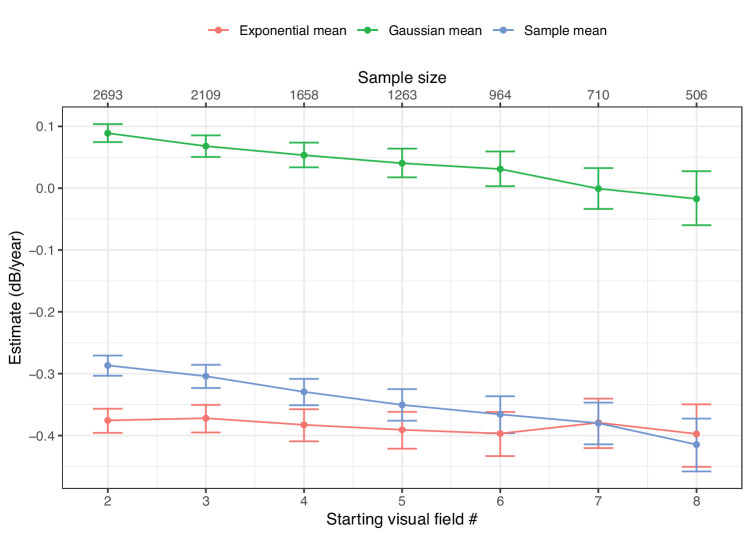
Estimated average value for the mean of the Gaussian distribution, the exponential distribution, and the combined mean of the exGaussian distribution (sample mean) describing the sample rate of progression for progressively trimmed series. The error bars indicate the 95% credible intervals.

Figure 4 shows how the offset in the mean of the Gaussian noise decreases by progressively eliminating earlier tests in the series, indicating that it is likely capturing the effect of learning. In contrast, the estimate of the “true” average rate of progression (the mean of the exponential distribution) remained relatively unaffected. The observed mean RoP (the same that would be measured with the standard Gaussian-LMM) is biased by the learning effect in the earlier tests. The Gaussian mean was effectively 0 dB/year at the seventh test.

**Figure 5.**
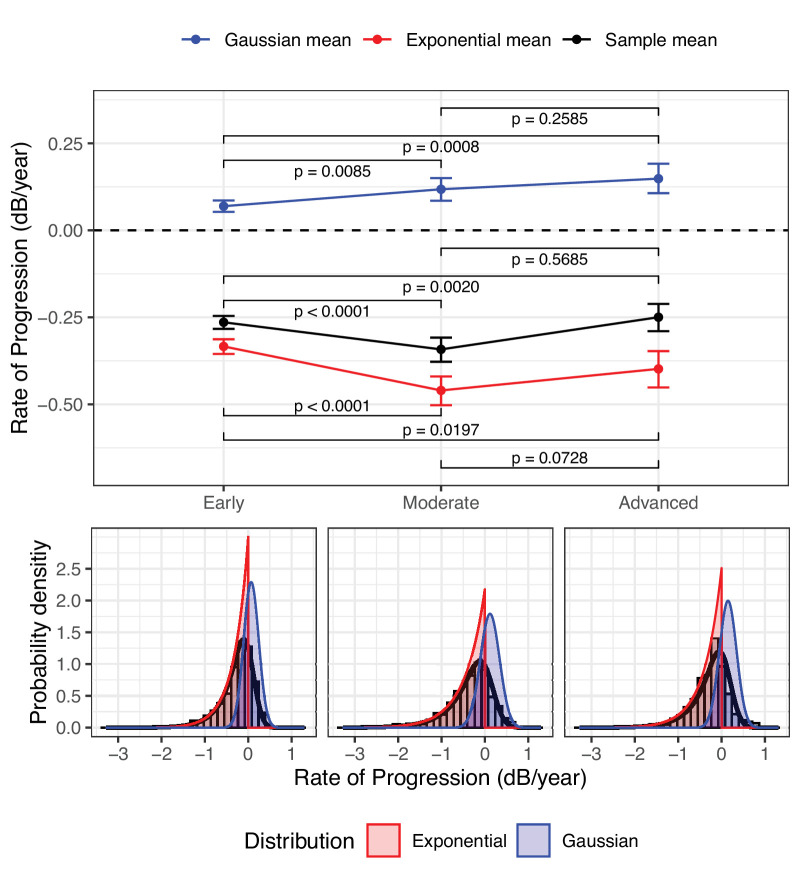
ExGaussian linear mixed model (LMM) parameter estimates (*top panel*) and slope distributions (*bottom panels*) in patients with estimated early, moderate, and advanced baseline perimetric loss. The sample mean is the sum of the exponential and gaussian means, equivalent to the estimate from a Gaussian-LMM (see Figure 3). Significance is expressed as a Bayesian *P* value (see Appendix for details on its calculation). The error bars indicate the 95% credible intervals. The estimated values are reported in the Supplementary Material.

Figure 5 shows a comparison of the ex-Gaussian parameter estimates obtained in the three different baseline severity groups. The mean of the Gaussian component (probable learning effect) was significantly more positive in the groups with moderate and advanced estimated baseline damage, compared to those with early loss. This resulted in a biased mean RoP, which was more positive in the advanced group than in the early group. In contrast, because it was not affected by learning, the estimated “true” rate was significantly more negative in the groups with moderate and advanced loss.

### Statistical Power in the Simulated Randomized Clinical Trials

**Figure 6.**
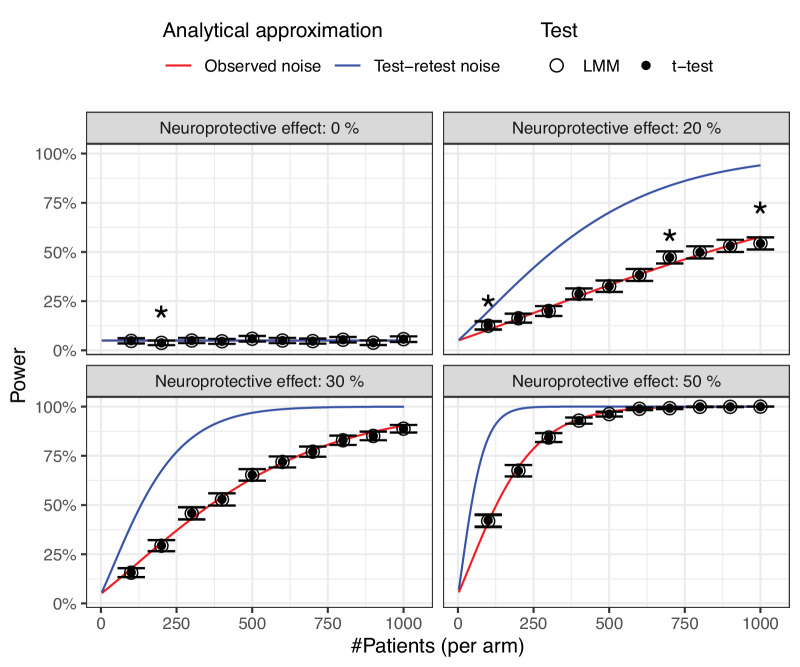
Power curves for simulated neuroprotection randomized controlled trials for different sample sizes and neuroprotective effects. The error bars indicate the 95% credible intervals for the simulated linear mixed model results. The solid curves are the analytical predictions using observed noise (*red*) and test-retest noise (*blue*) data. The *asterisks* indicate the cases where the prediction using observed noise data was outside the 95% CI. Untreated exponential rate of progression = −0.38 dB/year.

Figure 6 shows the power for the LMM and two-sample *t*-test on the empirical slopes in our simulated RCTs, for different neuroprotective effects and increasing sample sizes. The untreated “true” RoP was −0.38 dB/year (see the Table). The average residual SE of the MD was 1.97 dB. There was excellent agreement between the LMM and the *t*-test, with the largest absolute difference in power being 0.1%. Disagreement in determining a significant effect was observed only in 16 of 40,000 simulations (0.04%).

The analytical prediction was also in excellent agreement with the simulations, with a mean absolute difference in power of 1% (SD = 0.9%). The largest absolute difference was 3.4%. These results were identical with the LMM and the *t*-test. The analytically predicted values were outside the 95% credible intervals (CIs) of the simulated values only in 4 simulations, one when simulating no effect, where the simulations resulted in a power lower than the expected 5% false positive rate, and 3 when simulating the 20% neuroprotective effect. These are highlighted in Figure 6. We also report a comparison with the power curves estimated with the residual SE estimated from test-retest data (see Supplementary Material), which are more likely to be reflective of test noise in controlled environments, such as those of RCTs.

## Discussion

In this work, we provide a modeling approach to estimate the underlying distribution of RoPs, isolating the effect of noise and learning. We made the results of our modelling readily available to other researchers via a user-friendly web application. The estimated distribution shows good fit to the data, while providing fully interpretable parameters. This is in contrast with previous attempts which mainly tried to capture the features of the empirical distribution of RoPs,^9^^,^^10^^,^^12^ particularly their skewness. This has important consequences. In terms of clinical interpretation, it shows that noise and learning can effectively mask the true rate of VF progression. However, this method allows the estimation of a plausible underlying distribution of “true” rates in actual clinical populations. This is important for the derivation of accurate population parameters. For example, Figure 4 shows how the average rate of progression would be biased by the effect of learning in a naïve calculation. Our interpretation of the parameters is supported by specific results in our analysis. In particular, progressively taking out the initial tests in the series showed that the mean of the Gaussian component of the estimated *exGaussian* distribution (see Fig. 4) is likely modeling the average effect of learning. This effect came very close to 0 dB/year at the seventh test. This is in agreement with previous investigations,^13^ which estimated that the effect of learning can extend to the sixth or seventh VF. The effect of learning on population estimates is also clearly demonstrated in our analysis by severity group (see Fig. 5). We show that the effect of learning, and the consequent bias on the average rate of progression, is stronger in patients with a more advanced baseline MD. One explanation for this result is the overestimation of baseline damage in series affected by a strong learning effect or regression to the mean, because patients who performed the worst in their first tests were more likely to be classified as having moderate or advanced baseline loss. Despite this, the “true” estimated RoP was significantly faster in patients with moderate or advanced baseline loss compared to patients with early damage, as expected. However, the difference was not significant between patients with moderate and advanced damage. This was likely due to inaccurate classification of baseline damage (again due to learning affecting the initial tests) and the perimetric floor effect in truly advanced patients, which can positively bias the RoP of global metrics.^22^

The exponential distribution of rates estimated with the *exGaussian* distribution also offers straightforward physiological interpretations in the context of the progression of the disease, because it avoids the need to assume that the true rate of progression could be positive. Finally, the *exGaussian* distribution effectively captures the skewness in the data and can be used in hierarchical models instead of the commonly used Gaussian distribution, reducing the effect of shrinkage towards the mean (see Supplementary Material). Such a shrinkage can bias the trajectory of fast progressing eyes toward the general trend of the population, especially in those for which a smaller number of VF tests is available. This is supported by the improvement in WAIC compared to a Gaussian-LMM and the lack of significant departure of the empirical CDF of the RoPs from the estimated *exGaussian*, as shown by the KS test (*P* = 0.108). Importantly, such a model requires the estimation of the same number of parameters as a Gaussian-LMM, as described in the Methods section. This is also another important difference from previous attempts using different skewed distributions, which instead required the skewness to be modelled with an additional parameter.^11^

Such a characterization of the distribution of the RoPs is also extremely advantageous in the design of RCTs for glaucoma treatments, especially neuroprotection. Defining the effect of neuroprotection has always been challenging, because treatment effects are often defined as percentage change. However, the effect on VF RoPs is rarely described by a simple shift in the distribution of the untreated rates. Previous attempts have simulated a neuroprotective effect with a variety of methods, ranging from halting the progression of a proportion of patients^2^ to simply adding a positive effect to the observed RoPs to change the average rate by a predefined amount.^4^ Modeling the underlying “true” rate with a negative distribution, such as a negative exponential, offers a univocal definition of percentage change in RoPs by simply scaling, with a multiplicative factor, the “true” distribution. This not only replicates the complex distributional change in the observed RoPs that would be observed with treatment, but also affects the individual rates in a manner that replicates the underlying physiological process. Moreover, in the absence of learning (Gaussian noise mean = 0), a proportional change in the “true” exponential distribution also results in the same proportional change of the resulting *exGaussian* distribution. It is interesting to note that this is not true in the presence of learning. For example, in an RCT, the learning effect is likely to be the same in both arms. Therefore, the average difference in RoP, calculated, for example, with standard LMMs, would only depend on the average difference between the “true” RoPs (exponential). However, learning can positively bias the average RoP in both arms, and the same linear difference can result in various calculated “proportional” effects. This creates inconsistencies in trial design and in the interpretation and generalizability of the results, especially when naïve patients are recruited. For example, the treatment arm of the United Kingdom Glaucoma Treatment Study (UKGTS), in which true progression was likely to be slower, showed a positive median RoP (+0.12 dB/year), indicating a strong learning effect.^39^ Modeling an *exGaussian* distribution eliminates such an ambiguity because the linear difference in the average RoPs always corresponds to the same proportional change in the “true” distribution of rates.

This principle has been applied for our simulation experiments (see Fig. 6). It is interesting to note that the power curves produced by these simulations are in good agreement with those presented in a previous paper, based on the same population.^4^ Moreover, the *exGaussian* distribution makes it straightforward to calculate the expected variance and mean of the empirical distribution of RoPs based on the expected test variability, length of the follow-up and planned testing schedule. This allows a simple analytical approximation of the results of standard LMMs with a two-sample *t*-test, as explained in the Methods section and demonstrated in Figure 6, and makes it possible to translate our results into a user-friendly web application (https://giovannimontesano.shinyapps.io/Sample_size/). One important aspect to highlight is that the average residual SE estimated with linear regression from our sample was 1.97 dB, larger than the values reported in previous literature (closer to 1 dB).^34^^,^^40^ This discrepancy is possibly explained by the fact that our sample is composed of real-life test series over a long period of time (≥ 10 years). On the one hand, therapeutic intervention might have introduced deviations from a simple linear trend over such a long follow-up. On the other hand, suboptimal testing conditions might have created larger test fluctuations, which would be lower in more controlled testing conditions, such as those of test-retest variability studies or prospectively planned data collections. Such controlled testing conditions are more likely to reflect the perimetric noise that would be found in RCTs. We provide, as Supplementary Material, an in-depth analysis of the combined data from two perimetric test-retest datasets.^22^^,^^39^^,^^41^^,^^42^ The average test-retest SD from these datasets of MD was 0.94 dB, much closer to the literature, and was larger for patients with more advanced damage, as expected. Power calculations using these estimates are much more conservative and more likely to be appropriate for trial scenarios; these have been integrated in our web application and reported in Figure 6.

The exponential distribution for the “true” RoPs, combined with Gaussian noise, seems to accurately describe the distribution of rates observed in this large population. It should be noted that, for the *exGaussian* to effectively describe the observed data, the VF series need to be relatively homogenous in terms of number of tests and duration of follow-up. This is because series with different characteristics are, by definition, not sampled from the same distribution, because they would have different expected variance for the noise component (the SE of the slope). However, the *exGaussian*-LMM does account for such a heterogeneity in the estimation process, because the model calculates the expected SE of the slope based on the specific test series for each eye. This means that, in fact, each random slope is drawn from its own distribution, while simultaneously allowing the estimation of population parameters of interest. Naturally, there are cases where an exponential distribution might not reflect the underlying distribution of “true” rates. However, the exponential distribution seemed to provide a good fit for the observed data and allowed us to estimate an *exGaussian*-LMM with the same number of parameters as a simple Gaussian-LMM. Moreover, when testing a more general Gamma distribution in preliminary fitting experiments, we found that the shape parameter converged to a value slightly smaller than 1, making the exponential distribution a good candidate for our implementation (a Gamma distribution with a shape parameter of 1 is a simple exponential). Another example is provided by a recruitment strategy for a hypothetical trial which selects participants using cutoffs on the RoP measured in the clinic. We provide a description of the changes that such a selection would bring to the “true” distribution of RoPs, assumed exponential, in the Appendix. The resulting distribution would be extremely difficult to model exactly in any practical implementation of the LMM. However, we also show that, for the typical level of perimetric variability and a realistic number of tests that would be available in the clinic (4 to 6), the noise in the observed RoPs is such that, in practice, the resulting “true” distribution would only deviate minimally from the original exponential. In other words, such a selection would be largely ineffective in identifying patients truly progressing at a rate within a desired range. Note that this result is valid regardless of the distribution assumed for the “true” RoPs, because it is simply a consequence of the uncertainty affecting the estimation of the true rates from the observed rates.

Our approach did not specifically focus on modeling the intercepts. Although the overall shape of the distribution is very similar to that of the observed slopes, the exact distribution of the intercepts is not expected to be an *exGaussian*, as explained in the Appendix. Modeling the expected distribution under a simulated scenario is relatively straightforward (see Appendix). However, estimating all the parameters for such a distribution from clinical data would be challenging and very likely imprecise. The estimation of a plausible distribution becomes achievable with some simplifications and assumptions, but, in our analyses, it did not provide any improvement in fitting the data over assuming an *exGaussian* distribution for both intercepts and slopes (based on the WAIC, see Appendix). It should be noted that various choices of the distribution for the intercept, including a simple Gaussian, only had a minimal effect on the estimated distribution of the slopes, the actual parameter of interest.

It should be highlighted that the proposed modeling approach would not be restricted to VF progression, but to any metric the change in which is modeled with a linear trend over time and the “true” rate of which is not expected to be positive. This would be the case, for example, for optical coherence tomography measurements of the circumpapillary retinal nerve fiber layer or inner retinal macular layer thickness in glaucoma. This would give the chance to further test the assumptions related to the interpretation of the mean of the Gaussian component, because structural metrics are not expected to be affected by patients’ learning.

The current implementation of the model does not allow the estimation of the “true” RoP for each eye. This is because the individual RoP is modeled as the sum of a random draw from the estimated exponential and Gaussian distribution. This means that any combination of two real values drawn from these distributions and the sum of which is equal to the observed RoP is equally plausible. Importantly, although it might seem plausible to estimate individual learning at least for a series with clearly positive trends, it should be noted that such positive slopes can arise simply because of noise in the absence of learning, as shown in our simulations in Figure 1. Another limitation of the current implementation is that the model calculates the amount of noise based on the “average” level of test variability, independently of the RoP and level of damage. This is similar to standard implementations of hierarchical LMMs^22^ and facilitates comparison with standard methodology. Individual variability could be incorporated in the model, for example, by accounting for the level of damage.^34^ However, global indices such as MD are also largely influenced by global fluctuations in the performance,^4^^,^^34^^,^^35^ which are often subject-dependent. This means that both a systematic and a subject-level element of variability would need to be modeled, considerably increasing the complexity of the model and the number of parameters required. This will be the objective of future work. However, the estimated distribution appeared to accurately describe the observed distribution of RoPs calculated via linear regression (see Fig. 3).

Our modeling only focused on a global index, the MD. Although extensions to model pointwise values are relatively straightforward for Gaussian-LMMs,^22^ they pose significant challenges when implementing larger hierarchical structures for more complex distributions, such as in *exGaussian*-LMMs. Further extension of the model will focus on modeling an additional level in the hierarchy, for example, by modeling the RoP at each location and VF cluster.^22^^,^^27^^,^^28^ This is important for RCTs, because pointwise modeling has been beneficial in some retrospective analyses of trial data to highlight subtle differences^27^^,^^29^ in progression. However, a better characterization of fast progressors with the *exGaussian*-LMMs based on MD might prove sufficient to overcome the limitations of commonly used Gaussian-LMMs and provide similar power to pointwise LMMs. Other methods of analyzing RCT data, such as those based on machine learning,^43^^–^^45^ might also provide additional statistical power. However, such empirical techniques have the disadvantage of providing results that are often not directly interpretable. In contrast, our methodology was designed to provide estimates for parameters that had a clear interpretation in terms of disease progression, measurement noise and learning artifact, making it valuable in elucidating the actual impact on disease modification and hopefully improve translation of the results across different studies and datasets.

Finally, the translational effort of the current work focuses on exploiting our results to optimize the design of trials based on standard LMMs. Naturally, the *exGaussian*-LMM itself could be used for trials to estimate the change in the “true” RoP due to treatment. Although this could be easily implemented for individual trials, sample size calculations would be complicated due to the complex and time-consuming procedure required to estimate the parameters of the *exGaussian*-LMM. This is mainly because of the relatively complex, but fully interpretable, structure of the model to separate the parameters of the exponential and Gaussian component of the distribution. Defining approximate solution for power calculations with the proposed model will be the objective of future work.

## Supplementary Material

Supplement 1

## Figures and Tables

**Figure A1.**
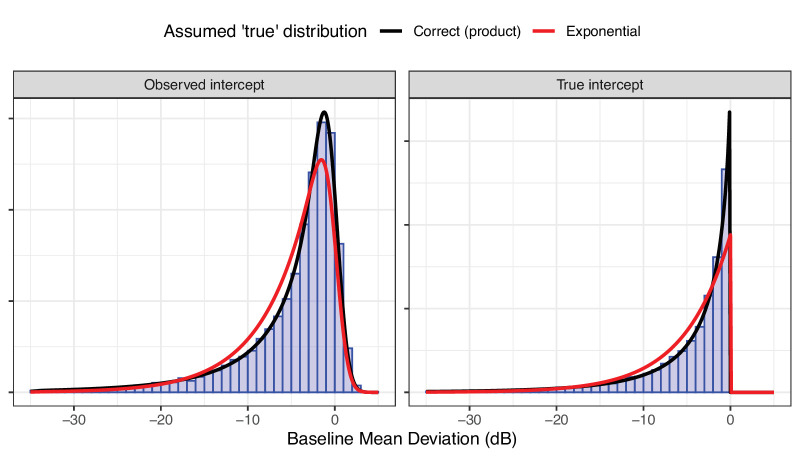
Distribution of intercepts from simulated test series, assuming an exponential distribution for the “true” rates of progression and a Gamma distribution for the interval between the insurgence of glaucoma and the first test.

**Figure A2.**
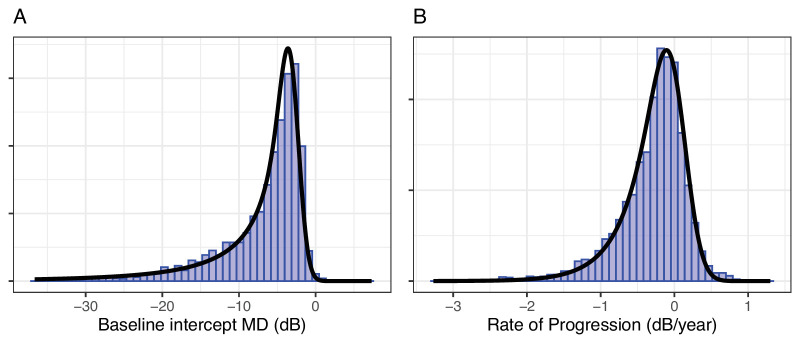
Fitting results for the intercept (**A**) and slope (**B**) using a linear mixed model using an exponential distribution for the “true” slope and an exponential-gamma product for the “true” intercept.

**Figure A3.**
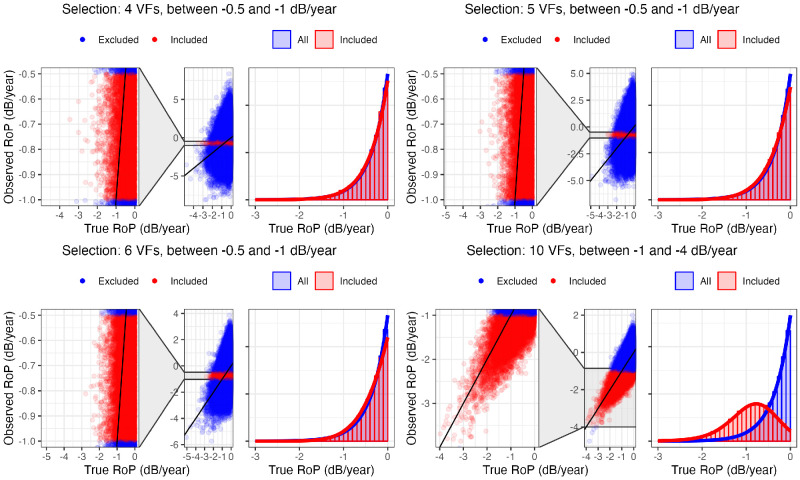
Distribution of the “true” rate of progression (RoP) of subsets of patients selected with cutoffs on their observed RoP, with various numbers of pre-trial visual field tests (VFs), performed every 6 months. The original distribution of “true” rates is exponential (*blue*). The distribution for the selected subset is in *red*. The histograms and scatterplots are generated with simulated data. The distribution for the selected subset is calculated as explained in the last paragraph of this Appendix.

## Appendix Appendix

### Bayesian Hierarchical Modeling

The Bayesian hierarchical LMMs were fitted by running Markov Chain Monte Carlo (MCMC) simulations in JAGS.^24^ The LMMs modelled a linear relationship of MD over time. There were two levels in the hierarchy, the population level and the subject level. The hierarchical LMM modelled individual progression by using random intercepts and slopes. We will describe the implementation of the standard Gaussian LMM and then explain how this was modified to use an *exGaussian* distribution for the random effects on the individual slopes.

The Gaussian-LMM estimates four population parameters for two Gaussian distributions of the random effects, that is, the mean and SD for the distribution of the intercepts (µ_0_ and σ_0_) and the slopes (µ_1_ and σ_1_). The individual intercepts and slopes where random draws from these Gaussian distributions. The residuals error of the predicted MD had a Gaussian distribution with mean 0 and SD σ_e_ (the residual SE). Note that, in standard LMMs, the random intercepts and slopes are usually modeled as a joint bivariate Gaussian distribution, to model the correlation between intercepts and slopes. We chose to instead model independent Gaussian distributions for the random effects to allow a direct comparison with the *exGaussian*-LMM, where the joint modeling is not easily implemented.

The *exGaussian*-LMM had a similar structure to the Gaussian-LMM. The difference was in the distribution of the random effects, which followed an *exGaussian* distribution. In this case, the random intercepts and slopes were considered to be the sum of a random draw from a negative exponential distribution and a Gaussian distribution (the noise component). The model estimated two population-level parameters for the *exGaussian* distribution, the parameter λ for the exponential distribution and the mean (µ_n_) of the Gaussian noise component. The SD σ_n_ of the noise was calculated as the SE of the intercept ($\sigma_{{\hat{\beta }}_{0}}^{2}$) and slope ($\sigma_{{\hat{\beta }}_{1}}^{2}$) defined as below (*t* indicates the time vector, *n* is the number of VF tests):

$$\begin{array}{c} \sigma_{{\hat{\beta }}_{0}}^{2}=\sigma_{e}^{2}\left[\frac{1}{n}+\frac{{\bar{t}}^{2}}{\sum_{i=1}^{n}{\left(t_{i}-\bar{t}\right)}^{2}}\right] \end{array}$$

$$\begin{array}{c} \sigma_{{\hat{\beta }}_{1}}^{2}=\frac{\sigma_{e}^{2}}{\sum_{i=1}^{n}{\left(t_{i}-\bar{t}\right)}^{2}} \end{array}$$

Note that each random intercept and slope was the sum of two draws from two independent random processes. This means that, although it is possible to estimate the population level parameter for the “true” exponential distribution, it is impossible to calculate the “true” rate of each subject, because there are infinite combinations of two values from the two distributions that can sum to give the same observed slope. One limitation of this method is that it estimates the overall σ_e_ rather than estimating the SE for each subject. This also means that the method assumes all subjects to have the same “average” level of variability. Although this is a common assumption in LMMs, it might not be reflective of the data.^25^ However, our results show that such an approximation does not have a large impact on the accuracy of our estimates (see Fig. 6). The model could be further expanded, in the future, to account for changes in variability due to the level of damage or individual variations, if this is required for specific contexts.

The prior distributions for the mean of the random effects of the Gaussian-LMM were normal distributions, with mean = 0 and SD = 100 for the intercepts and SD = 10 for the slopes (units are in dB and dB/year, respectively). The prior distributions for the variances of the random effects were both set as weakly informative Inverse-Gamma with shape and rate parameters equal to 0.001. Two parallel MCMC chains were run for 10,000 iterations, with 5000 additional burn-in iterations. The Gelman-Rubin index was assessed to ensure convergence (GR < 1.2).^46^

The prior distributions in the *exGaussian*-LMM were set as partially informative for the main calculations. The prior distribution for the parameter λ was a Gamma distribution with shape 1 and rate 1/$\hat{\lambda }$. The value of $\hat{\lambda }$ was calculated using the method of moments for the *exGaussian* distribution^47^ from the empirical values of intercepts and slopes obtained through linear regression. This Gamma prior has mean = $\hat{\lambda }$ and variance = 1/${\hat{\lambda }}^{2}$. Note that whereas the method of moments can be used to estimate the parameters of an *exGaussian* distribution and can provide useful prior information, it would fail to establish a link between the observed values and the underlying generating random process, especially for the RoP. The fitting procedure was identical to the Gaussian-LMM.

We used the WAIC to compare the two models. This was calculated by monitoring the log-likelihood of the observations during the fitting process for the MCMC draws. These values were later used to calculate the WAIC (and its SE) for the Gaussian and *exGaussian*-LMM using the *loo* package for R.^30^ The package also provides the SE for the difference in WAIC (lower WAIC indicates a preferrable model). As previously explained, because the WAIC can be influenced by the choice of priors, we compared the Gaussian-LMM to an *exGaussian*-LMM fitted with similarly weakly informative priors. Specifically, the prior distribution for the parameter λ was a Gamma with shape and rate 0.001 (the same as the distribution for the inverse variance of the random effects in the Gaussian-LMM).

### Modeling the Distribution of the Intercepts

As explained in the Methods section, the “true” distribution of the intercepts, assuming an exponential distribution of the RoPs prior to the first test, is the product of the exponential distribution of RoPs and the time interval between the development of glaucoma and the first VF test. The time interval can potentially have any strictly positive distribution. The Gamma is a positive distribution with two parameters (shape and rate) that would offer sufficient flexibility to model a variety of scenarios. Note that the exponential distribution is a special case of Gamma distribution with shape = 1. The PDF of the product of two Gamma distributed random variables (or a Gamma and an exponential) has the analytical expression reported in the last paragraph of this Appendix. In the example below, we sampled from an exponential with rate λ = 1.12 and a Gamma with shape 2 and rate 0.34 (which corresponds to an average time interval of 6 years). The product distribution is still single-tailed and can be approximated with an exponential with mean equal to the product of the means of the two distributions (the mean for the exponential distribution is 1/λ). The correct distribution has, however, a faster decaying tail (Fig. A1), which is consistent with the distribution observed for the empirical intercepts in our dataset (see Fig. 3).

The parameters for the product distribution can be estimated with a hierarchical LMM similar to the others in this study by making the simplifying assumption that the time to detection is also described by an exponential distribution (i.e. by setting the shape parameters of the Gamma distribution to 1) and that the distribution of the “true” RoPs before the baseline test is the same as the one estimated from the follow-up. Note that this is unlikely to be true because patients would be managed to reduce their RoP during follow-up. This approach models the distribution of the intercept as the sum of a Gaussian distribution and a product of two exponential distributions. The results are presented in Figure A2. However, the WAIC with this model was not better than the full *exGaussian*-LMM (192197.5 [725.7]).

### Statistical Comparison of Parameter Estimates

The Bayesian *P* value was calculated according to Makowski et al.^32^ The median of the posterior distribution is determined from the MCMC draws. According to whether the median is above or below 0, the proportion of MCMC draws above or below 0 is the *P*-direction, respectively. The *P*-direction can have values between 0.5 (distribution centered on 0) and 1. The Bayesian *P* value statistic can then be calculated as p = 2 * (1–*P-direction*) ^32^ and has a similar interpretation to a two-tailed frequentist *P* value.

### Effect of Selective Sampling

Trial patients can be selected based on their observed RoP in the clinic prior to recruitment. This would modify the expected exponential distribution for the “true” RoPs. This situation can be easily simulated by selectively rejecting observed RoPs above or below a desired cutoff. A simple analytical expression for the resulting distribution does not exist. However, it can be very efficiently evaluated numerically. For a single cutoff, for example, to select people with an observed RoPs more negative than −0.5 dB/year, the unscaled PDF can be obtained by multiplying the PDF of the exponential distribution of “true” RoPs by the CDF of a Gaussian distribution with mean −0.5 dB/year and SD equal to the average SE of the slopes measured from clinically collected data. The resulting curve needs to be normalized so that it integrates to 1 to obtain a valid PDF. Simulated examples are shown in Figure A3. It is useful to evaluate the practical impact of such a selection. Assuming an interval between tests of 6 months and an average $\sigma_{e}^{2}$ = 3.87 dB, we can estimate the values of $\sigma_{{\hat{\beta }}_{1}}$ for the RoPs estimated from the last 4, 5, or 6 tests prior to recruitment. These values are 1.76 dB/year, 1.24 dB/year, and 0.94 dB/year, respectively. The result of selecting patients with an observed RoP between −0.5 dB/year and −1 dB/year are shown in Figure A3. Because of the large variability of the observed slopes for the number of tests that would likely be available from the clinic in most patients, the selection has little effect on the distribution of “true” rates. A much higher precision and stronger selection criteria would be required to meaningfully affect their distribution (see Fig. A3).

### Relevant Formulas

#### ExGaussian Distribution

Probability distribution function (PDF):

$$\begin{array}{c} f\left(x|\lambda ,\mu ,\sigma \right)=\frac{\lambda }{2}e^{\frac{\lambda }{2}\left(2\mu +\lambda \sigma^{2}-2x\right)}\Phi \left(\frac{x\;-\;\mu +\lambda \sigma^{2}}{\sigma }\right) \end{array}$$

Cumulative distribution function (CDF):

$$\begin{array}{ccc} F(x|\lambda ,\mu ,\sigma ) & = & \Phi \left(\frac{x\;-\;\mu }{\sigma }\right)-\;\Phi \left(\frac{x\;-\;\mu +\lambda \sigma^{2}}{\sigma }\right)\\ & & \times \;e^{\left(\frac{{\left(\mu +\lambda \sigma^{2}\right)}^{2}-\mu^{2}-2x\lambda \sigma^{2}}{2\sigma^{2}}\right)} \end{array}$$

Where Φ indicates the CDF of a standard Gaussian distribution. λ indicates the rate of the exponential component. µ and σ indicate the mean and standard deviation of the Gaussian component. The PDF of the *exGaussian* distribution is obtained as the convolution of the Gaussian PDF

$$\begin{array}{c} f\left(x|\mu ,\sigma \right)=\;\frac{e^{-\frac{1}{2}\;{\left(\frac{x-\mu }{\sigma }\right)}^{2}}}{\sigma \sqrt{2\pi }} \end{array}$$

and the Exponential PDF

$$\begin{array}{c} f\left(x|\lambda \right)=\;\lambda e^{-\lambda x}. \end{array}$$

#### Product of Gamma and Exponential

This models the distribution of “true” intercepts (baseline MD) assuming an exponential distribution of RoPs prior to the first test, a Gamma distributed interval between the development of glaucoma and first test and that all subjects have an MD = 0 dB before developing glaucoma.

$$\begin{array}{c} f\left(x|\alpha ,\beta_{1},\beta_{2}\right)=\frac{2{\left(\frac{x}{\beta_{1}*\beta_{2}}\right)}^{\left(\frac{\alpha +1}{2}\right)}BesselK\left(2\sqrt{\frac{x}{\beta_{1}*\beta_{2}}},\alpha -1\;\right)}{\Gamma \left(\alpha \right)x} \end{array}$$

β_1_ and β_2_ are the inverse of the rates for the exponential and the Gamma distribution, α is the shape parameter of the Gamma distribution. *BesselK* indicates a Bessel function of the second kind. These functions can be complex to calculate, but are implemented in most languages, including base R. The effect of perimetric noise and intersubject variability of MD can be modelled with a Gaussian distribution. Similarly to the modeling of the slope, the distribution for the observed intercepts can be obtained by convolving the “true” distribution of the intercepts with this Gaussian distribution. In the case of a simple exponential, the result is an *exGaussian*. The convolution with the PDF reported above has no close form solution and can be obtained via numerical convolution.

#### Effect of Patient Selection

The unscaled PDF of the “true” rates after selecting patients with an observed rate below a certain threshold *c*, with an estimated standard error for the observed rates $\sigma_{{\hat{\beta }}_{1}}$, is:

$$\begin{array}{c} f\left(x|\lambda ,c,\sigma_{{\hat{\beta }}_{1}}\right)\;=\;\lambda e^{-\lambda x}\left(1\;-\;\Phi \left(\frac{x\;-\;c}{\sigma_{{\hat{\beta }}_{1}}}\right)\right) \end{array}$$

The opposite selection (i.e. patients with an observed rate above a certain threshold *c*) can be calculated as:

$$\begin{array}{c} f_{u}\left(x|\lambda ,c,\sigma_{{\hat{\beta }}_{1}}\right)\;=\;\lambda e^{-\lambda x}\Phi \left(\frac{x\;-\;c}{\sigma_{{\hat{\beta }}_{1}}}\right) \end{array}$$

These unscaled PDFs need to be normalized to integrate to unity to represent a valid PDF. This can be achieved numerically by calculating the normalization factor as integral of the unscaled PDF:

$$\begin{array}{c} f\left(x|\lambda ,c,\sigma_{{\hat{\beta }}_{1}}\right)\;=\frac{f_{u}\left(x|\lambda ,c,\sigma_{{\hat{\beta }}_{1}}\right)}{\int_{-\infty }^{\infty }f_{u}\left(x|\lambda ,c,\sigma_{{\hat{\beta }}_{1}}\right)dx} \end{array}$$

Note that the selection can be applied recursively to select patients within a certain interval.
