# Supplementary material for: Estimating the Distribution of True Rates of Visual Field Progression in Glaucoma
Source: Transl Vis Sci Technol. 2024 Apr 9;13(4):15. doi: 10.1167/tvst.13.4.15 (PMC11008752; doi:10.1167/tvst.13.4.15)
Supplement: Supplement 1 [file tvst-13-4-15_s001.pdf]

Supplementary to: Estimating the distribution of true rates of visual field progression in glaucoma

Giovanni Montesano[1,2], David F. Garway-Heath[2], David M. Wright[3], Alessandro Rabiolo[4,5], Giovanni Ometto[1,2], David P. Crabb[1]

1. City, University of London, Optometry and Visual Sciences, London, United Kingdom
2. NIHR Biomedical Research Centre, Moorfields Eye Hospital NHS Foundation Trust and UCL Institute of Ophthalmology, London, United Kingdom
3. Centre for Public Health, Queen's University Belfast, Block B, Royal Hospital, Belfast, Northern Ireland
4. Department of Health Sciences, University East Piedmont "A. Avogadro", Novara, Italy
5. Eye Clinic, University Hospital Maggiore della Carità, Novara, Italy

Modelling of test-retest variability

In our main analysis, the test-retest variability of Mean Deviation (MD) was inferred from the average residual standard error (SE), an unbiased estimate of residual standard deviation (SD, i.e. the measurement noise). However, our estimated residual SE (1.97 dB) was considerably higher than previous estimates from the literature[1, 2], which were closer to 1 dB. This can have a significant impact on power calculations.

In this supplementary appendix, we offer some estimates of MD variability based on short-term test-retest visual field (VF) data. We combined two datasets, which have been extensively described elsewhere[3-6].

The RAPID dataset[4, 5] is composed of 1,396 Humphrey Field Analyzer (HFA) 24-2 SITA-Standard tests from 146 eyes of 75 patients with glaucoma, who performed up to 10 tests per eye over 3 months (minimum of 3 tests for inclusion). The median number of test repeats per eye was 10 (5th, 95th percentile: 7, 10). Data were collected at Moorfields Eye Hospital (ethics reference 13/NS/0132) upon written informed consent. The data collection was in accordance with the Declaration of Helsinki.

The Halifax dataset[6] is freely available from the *visualFields*[7] package and is composed of 360 HFA 24-2 SITA-Standard tests from 30 eyes of 30 patients with glaucoma, who performed 12 tests over 3 months. The summary characteristics of the two samples are reported in **Table 1**. All tests in both datasets had a false positive error rate < 15%, the only criterion used to assess reliability[8, 9].

	RAPID Dataset	HALIFAX Dataset
**Age (years)**	70 [64, 76]	69 [64, 70]
**BCVA (logMAR)**	0 [-0.08, 0.18]	-
**SE (D)**	0 [-1.35, 0.88]	-
**IOP (mmHg)**	14 [12, 16]	-
**Average MD (dB)**	-3.29 [-7.76, -1.24]	-2.57 [-4.36, -1.45]
**Average PSD (dB)**	4.26 [2.16, 9.6]	3.11 [1.98, 5.39]

Table 1. Demographic information for the three datasets used in this study, reported as Median [Interquartile Range]. Average = patient-average calculations. BCVA = Best Corrected Visual Acuity; SE = Spherical Equivalent; D = Dioptres; IOP = Intraocular Pressure; MD = Mean Deviation; PSD = Pattern Standard Deviation.

We calculated the test-retest variance and average MD of each series. We then calculated the average standard deviation (SD) as the square-root of the average variance, because variance, but not SD, can be combined linearly (i.e. summed and averaged). The calculation was performed for the whole sample and for three stages of severity: Early (average MD ≥ -6 dB); Moderate (-6 dB > average MD ≥ -12 dB); Advanced (average MD < -12 dB).
We also modelled the continuous change in MD variability according to the level of damage. This was achieved with a generalised linear model (GLM), fitted in R (R Foundation for Statistical Computing, Vienna, Austria). The model used a logarithmic link function to estimate a continuous relationship (second degree polynomial) between average MD and variance. Note that, differently from standard linear models on log-transformed data, log-GLMs estimate a model for the logarithm of the mean rather than the mean of the logarithm. Therefore, the estimates can be un-logged to obtain the mean variance (and SD) for any value of average MD. The log-GLM also ensures that the predicted mean variance is always strictly positive.
The results are reported in **Figure 1**. The predictions from a model of the log-transformed variance are also reported for comparison.

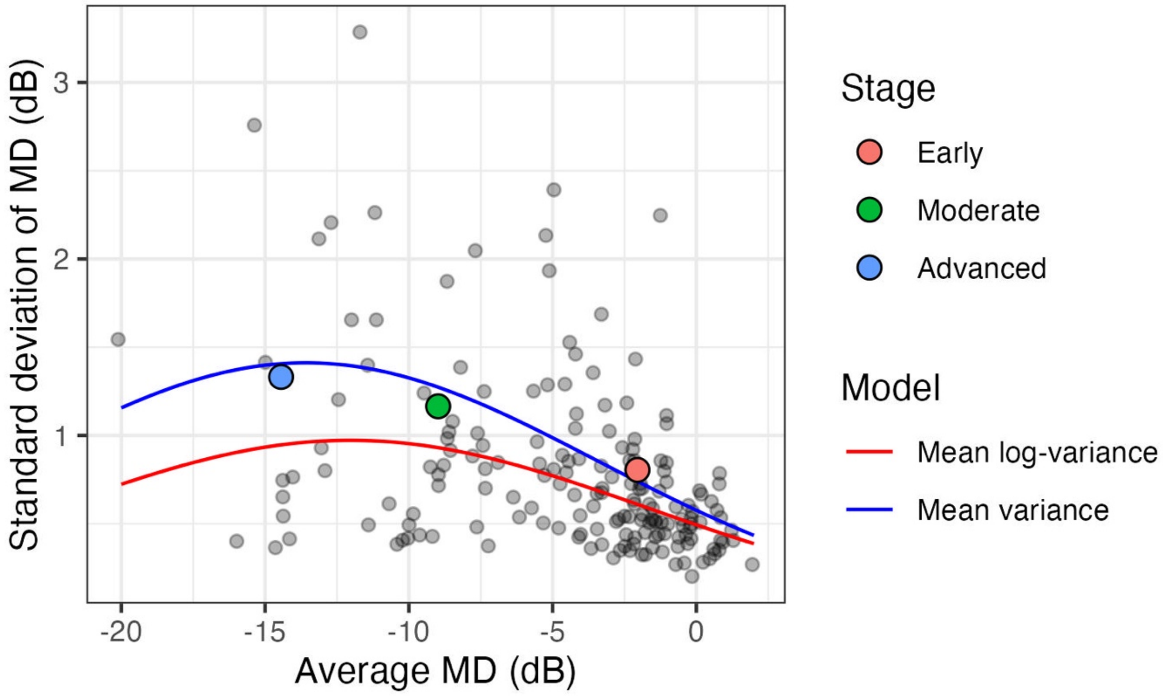

Figure 1. Modelling of test-retest variability of the Mean Deviation (MD). The plot shows how the raw stratified average standard deviations (SDs) are close to the SD predicted by the generalised linear model of the mean variance, but not to the estimates obtained from modelling the mean of the log-transformed variance.

The overall mean test-retest SD was 0.94 dB (**Early damage**: 0.80 dB; **Moderate damage**: 1.17 dB; **Advanced damage**: 1.33 dB). The estimated continuous model was:

$$SD_{dB} = \sqrt{exp(-1.102611 - 0.263774 * MD_{dB} - 0.009706 * MD_{dB}^2)}$$

All terms were statistically significant (largest p = 0.0215, for the quadratic term).

Watanabe-Akaike Information Criterion for increasing series length

Series length	WAIC [95%-Confidence Intervals]		
Number of tests	exGaussian	Gaussian	Difference
4	57517.0 [56739.4, 58294.7]	58459.3 [57764.0, 59154.6]	942.3 [737.1, 1147.5]
5	71724.2 [70887.2, 72561.3]	72539.3 [71760.9, 73317.7]	815.1 [640.3, 989.8]
6	86084.5 [85073.7, 87095.4]	86764.9 [85820.4, 87709.5]	680.4 [526.6, 834.2]
7	100015.8 [98902.7, 101128.8]	100646.4 [99596.0, 101696.8]	630.6 [499.9, 761.4]
8	114076.2 [112898.4, 115254.0]	114664.0 [113536.8, 115791.1]	587.8 [462.0, 713.5]
9	128587.5 [127356.1, 129818.9]	129172.9 [127983.0, 130362.8]	585.4 [451.8, 719.0]
10	143831.3 [142538.5, 145124.2]	144371.5 [143124.0, 145619.0]	540.2 [396.7, 683.6]
**All**	192174.4 [190760.8, 193588]	192595.0 [191228.1, 193962.0]	420.6 [264.4, 576.8]

Table 2. Watanabe-Akaike Information Criterion (WAIC) for series of increasing length, showing a statistically significant improvement with the exGaussian model for all series lengths (95%-Confidence Intervals of the differences do not include 0).

Shrinkage of random effect estimates

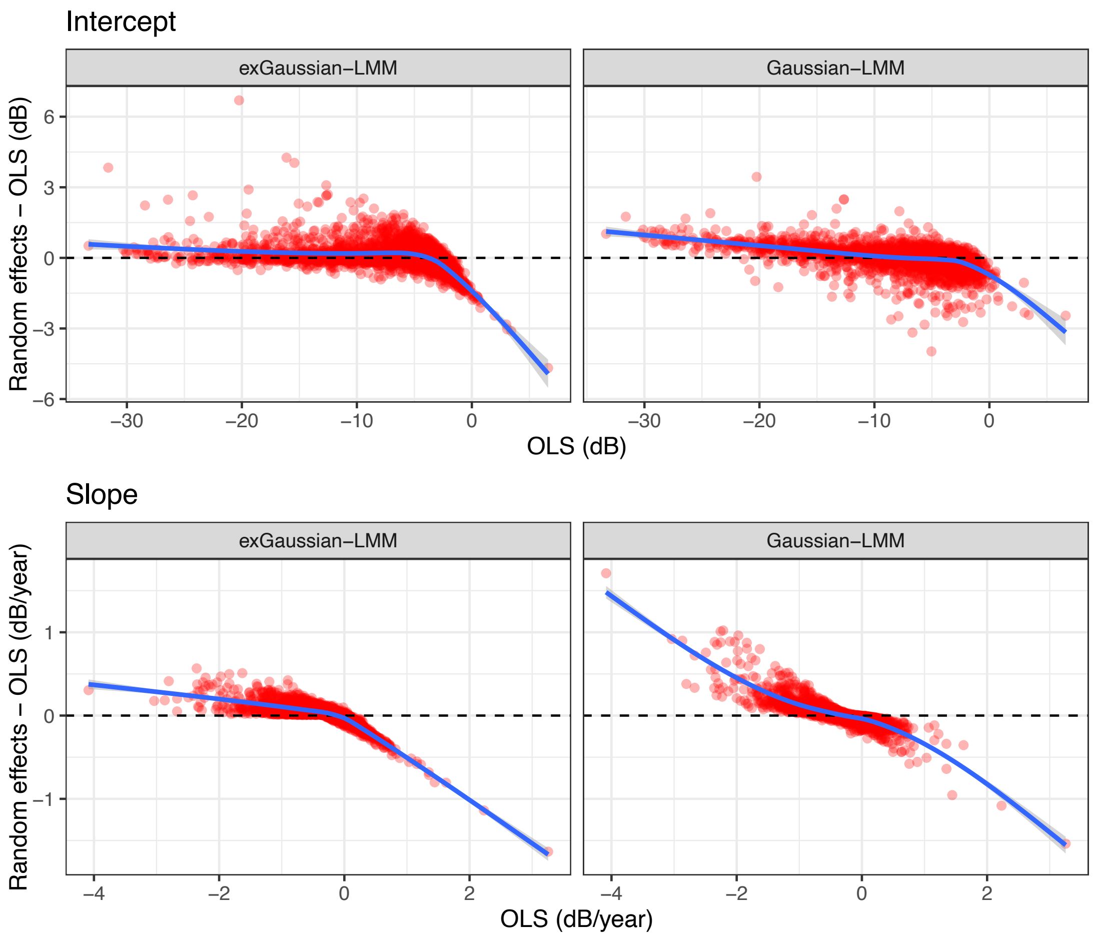

Figure 2. Comparison of intercept and slope estimates from random effects and ordinary least squares (OLS). The plots shows the difference between the estimates stratified by their OLS estimate. The Gaussian linear mixed model (LMM) introduces a stronger shrinkage towards the population mean for fast negative slopes compared to the exGaussian-LMM, potentially biasing the estimates.

Slope parameter estimates stratified by sensitivity

	ExGaussian-LMM		Gaussian-LMM
	Exponential mean (dB/year)	Gaussian mean (dB/year)	Sample mean (dB/year)
**Early**	-0.33 [-0.36, -0.31]	0.07 [0.05, 0.09]	-0.26 [-0.28, -0.25]
**Moderate**	-0.46 [-0.50, -0.42]	0.12 [0.09, 0.15]	-0.34 [-0.38, -0.31]
**Advanced**	-0.40 [-0.45, -0.35]	0.15 [0.11, 0.19]	-0.25 [-0.29, -0.21]

Table 3. Estimated model parameter values for the slope. The sample mean is the sum of the exponential and gaussian means and is equivalent to the mean estimated by the Gaussian-LMM. LMM = Linear Mixed Model. Early (baseline MD ≥ -6 dB); Moderate (-6 dB > baseline MD ≥ -12 dB); Advanced (baseline MD < -12 dB).

References

1. Wall M, Doyle CK, Zamba KD, et al. The repeatability of mean defect with size III and size V standard automated perimetry. Invest Ophthalmol Vis Sci 2013;54(2):1345-51.
2. Wu Z, Medeiros FA. Development of a Visual Field Simulation Model of Longitudinal Point-Wise Sensitivity Changes From a Clinical Glaucoma Cohort. Transl Vis Sci Technol 2018;7(3):22.
3. Montesano G, Garway-Heath DF, Ometto G, Crabb DP. Hierarchical Censored Bayesian Analysis of Visual Field Progression. Transl Vis Sci Technol 2021;10(12):4.
4. Garway-Heath DF, Quartilho A, Prah P, et al. Evaluation of Visual Field and Imaging Outcomes for Glaucoma Clinical Trials (An American Ophthalomological Society Thesis). Trans Am Ophthalmol Soc 2017;115:T4.
5. Garway-Heath DF, Zhu H, Cheng Q, et al. Combining optical coherence tomography with visual field data to rapidly detect disease progression in glaucoma: a diagnostic accuracy study. Health Technol Assess 2018;22(4):1-106.
6. Artes PH, O'Leary N, Nicolela MT, et al. Visual field progression in glaucoma: what is the specificity of the Guided Progression Analysis? Ophthalmology 2014;121(10):2023-7.
7. Marin-Franch I, Swanson WH. The visualFields package: a tool for analysis and visualization of visual fields. J Vis 2013;13(4).
8. Yohannan J, Wang J, Brown J, et al. Evidence-based Criteria for Assessment of Visual Field Reliability. Ophthalmology 2017;124(11):1612-20.
9. Bengtsson B. Reliability of computerized perimetric threshold tests as assessed by reliability indices and threshold reproducibility in patients with suspect and manifest glaucoma. Acta Ophthalmol Scand 2000;78(5):519-22.
